# Cyclophilin Inhibitors: An Emerging Class of Therapeutics for the Treatment of Chronic Hepatitis C Infection

**DOI:** 10.3390/v4112558

**Published:** 2012-10-26

**Authors:** Sam Hopkins, Philippe Gallay

**Affiliations:** 1 Autoimmune Technologies, LLC, 1010 Common Street, Suite 1705, New Orleans, LA 70112, USA; 2 Department of Immunology and Microbial Science, IMM-9, The Scripps Research Institute, 10550 N. Torrey Pines Rd., La Jolla, CA 92037, USA

**Keywords:** HCV, cyclophilins, cyclophilin inhibitors

## Abstract

The advent of the replicon system together with advances in cell culture have contributed significantly to our understanding of the function of virally-encoded structural and nonstructural proteins in the replication cycle of the hepatitis C virus. In addition, *in vitro* systems have been used to identify several host proteins whose expression is critical for supporting such diverse activities as viral entry, RNA replication, particle assembly, and the release of infectious virions. Among all known host proteins that participate in the HCV replication cycle, cyclophilins are unique because they constitute the only host target that has formed the basis of pharmaceutical drug discovery and drug development programs. The introduction of the nonimmunosuppressive cyclophilin inhibitors into clinical testing has confirmed the clinical utility of CsA-based inhibitors for the treatment of individuals with chronic hepatitis C infection and has yielded new insights into their mechanism(s) of action. This review describes the biochemical evidence for the potential roles played by cyclophilins in supporting HCV RNA replication and summarizes clinical trial results obtained with the first generation of nonimmunosuppressive cyclophilin inhibitors.

## 1. Introduction

The advent of the replicon assay has unquestionably expanded our knowledge of the hepatitis C virus (HCV) replication cycle [1]. In particular, the increased understanding of the function of virally‑encoded, nonstructural proteins has led to the identification of multiple new classes of Direct Acting Anti-HCV Agents (DAAs) for the treatment of individuals with chronic HCV infection [2]. These DAAs include the recently approved NS3/4A serine protease inhibitors Victrelis (boceprevir) and Incivek (telaprevir) and investigational agents including NS5A inhibitors as well as several diverse types of nucleoside/nucleotide and nonnucleoside inhibitors of NS5B, the RNA-dependent RNA polymerase. More recent advances in cell culture techniques including the use of soluble recombinant E2, HCV pseudo particles, and infectious cell culture-produced virions have enabled a better understanding of the function of virally-encoded nonstructural proteins and has led to the development of *in vitro* systems that support the complete HCV replication cycle [3]. These same advances have also led to the identification of a subset of host-encoded cofactors whose expression is essential in order to support virtually all aspects of the viral replication cycle. In several instances approved drugs have been used as prototypical inhibitors in order to vet these host proteins as potential targets for pharmaceutical intervention strategies. Anti-cancer agents were most recently used to identify the receptor tyrosine kinase activity associated with epidermal growth factor receptor and ephrin receptor A2 as potential host targets [4]. Inhibition of receptor tyrosine kinase activity by erlotinib or dasatinib prevented CD81 and claudin-1 co-receptor association and viral glycoprotein-dependent membrane fusion. At this time no receptor tyrosine kinase inhibitors have attained a clinical testing status. The importance of host lipid metabolizing enzymes was established by demonstrating the *in vitro* anti-HCV activity of lovastatin, a specific inhibitor of 3-hydroxy-3-methyl-glutaryl Coenzyme A reductase (HMG CoA reductase) and geranylgeranyl transferase I [5]. Exposure of replicon cells to lovastatin promoted disassembly of the replication complex, which was reversible upon addition of geranylgeraniol to the culture medium. Various members of the statin family have been assessed for their *in vitro* anti-HCV activity; however, the clinical utility of statins in the treatment of chronic hepatitis C infection remains controversial. Cyclosporine A (CsA) is a well-characterized immunosuppressive agent that inhibits the peptidyl-prolyl isomerase activity associated with the broad family of cyclophilins (Cyps) at nanomolar concentrations [6]. The complex formed between CsA and cyclophilin A (CypA) is a potent inhibitor of calcineurin. The formation of this ternary complex leads to inhibition of the intrinsic phosphatase activity of calcineurin, which abolishes the dephosphorylation-driven nuclear translocation of the nuclear factor of activated T cells (NFAT). This ultimately contributes to the failure of T cells to respond to antigenic stimuli. Early studies comparing the *in vitro* anti-HCV activities of CsA and NIM811 (a nonimmunosuppressive analog of CsA that binds to CypA, but lacks the ability to inhibit calcineurin phosphatase) established that antiviral effects were associated with binding to host CypA and were independent of inhibitory effects on calcineurin and were also independent of inhibitory activity towards the multi-drug resistance protein, P‑glycoprotein (P-gp) [7]. Further studies established that replication of HCV-specific RNA depends on the expression of CypA [8]. CsA was therefore used as the starting material for medicinal chemical programs in order to generate analogs that retained their CypA binding properties, but lacked the dose‑limiting calcineurin binding activity. SCY-635 and Alisporivir (DEB025) together with NIM811, which was isolated as a side product of CsA biosynthesis, now represent the most advanced of all host‑targeted antiviral agents. Clinical proof of concept has been established for all three compounds either as monotherapy [9,10] or when given in combination with pegylated interferon [11].

This review will focus on describing the discovery of Cyps as essential host co-factors that support HCV RNA replication, biochemical studies that identify the potential mechanism(s) of action for nonimmunosuppressive Cyp inhibitors, and clinical trial results obtained to date with all members of this emerging class of host-targeted antiviral agents.

## 2. Results and Discussion

### 2.1. Host Cell Expression of Cyclophilins Is Essential in Order to Support HCV RNA Replication for All Genotypes

The Shimotohno laboratory was the first to demonstrate that the immunosuppressive drug CsA efficiently suppresses HCV replication in cultured hepatoma cells [12]. This prime discovery was corroborated by subsequent studies, which showed that not only CsA, but non-immunosuppressive CsA derivates such as NIM811, Alisporivir and SCY-635 also block HCV replication [7,13,14,15,16,17]. Moreover, studies showed that sanglifehrins as well as polyketide sanglifehrin derivates also inhibit HCV replication [14,18,19,20]. Sanglifehrins are natural products that also bind Cyps, but are structurally distinct from CsA [19]. Since Cyps represent the main intracellular targets for CsA and sanglifehrins, the above findings strongly suggest a direct relationship between Cyps and HCV. Corroborating this hypothesis, transient knockdown studies indicated that multiple Cyps assist HCV [21,22]; then stable knockdown studies suggested that CypA is the main Cyp member, which governs HCV replication [8,22,23]. Further supporting that CypA is vital for HCV, the reintroduction of CypA into CypA‑knockdown cells restores HCV replication [8,22,23]. CypA was found to be associated with HCV replication complexes [24,25], suggesting that the host protein is expressed in the relevant biological compartment to support HCV. Altogether these data strongly suggest that HCV highly relies on host CypA to replicate in cells. 

CypA was originally discovered as a cellular ligand for CsA [26]. CypA is an abundant cytosolic protein that is expressed in all eukaryotic cells [26]. CypA is a peptidyl-prolyl *cis-trans* isomerase [27], which accelerates the *cis* to *trans* interconversion of proline-containing peptides or proteins [28]. CypA possesses a hydrophobic pocket that contains both the enzymatic and ligand binding sites of the protein [29]. CsA, sanglifehrins and their derivates, by binding within this hydrophobic pocket, neutralize both the enzymatic and ligand binding activities of CypA [19,20]. Although CypA has been shown to act as a peptidyl-prolyl *cis-trans* isomerase *in vitro*, its true cellular function remains to be demonstrated. Importantly, CypA-knockout mice and T cell lines are perfectly viable [30,31], suggesting that CypA represents an appropriate target for Cyp inhibitors (nonimmunosuppresive CsA and sanglifehrin derivates) in HCV patients.

### 2.2. Biochemical Studies Describing the Potential Mechanism(s) of Action of Cyclophilin Inhibitors

The antiviral mechanisms of action of Cyp inhibitors are not fully understood. However, several key findings provide putative mechanisms of action for these potent anti-HCV agents. The first key finding is that the isomerase pocket of CypA is critical for HCV replication [8,24]. Specifically, in contrast to wild-type CypA, isomerase-deficient CypA mutants, which harbor mutations in their hydrophobic pocket, are unable to support HCV replication [8,24,32]. This is in accordance with the fact that Cyp inhibitors neutralize the enzymatic activity of CypA by binding to its hydrophobic pocket [33,34]. The second key finding is that CypA binds directly to the HCV nonstructural 5A (NS5A) protein. Early studies nicely showed that HCV variants, which arose under Cyp inhibitor selection, developed mutations in the NS5A gene [14,18,35,36,37,38,39,40,41,42]. This led researchers to postulate that NS5A serves as a viral ligand for CypA. Several independent laboratories using various technologies (*i.e.*, NMR, recombinant and cellular pulldowns) convincingly demonstrated that indeed CypA and NS5A form complexes [20,35,37,39,42,43,44,45,46]. CypA-NS5A interactions are conserved among all HCV genotypes [37,42]. The third key finding is that Cyp inhibitors prevent and disrupt CypA-NS5A complexes. Several studies showed that all classes of Cyp inhibitors—CsA, nonimmunosuppressive CsA derivates (e.g., Alisporivir and SCY-635), sanglifehrins and sanglifehrin derivates—block CypA‑NS5A interactions in a dose-dependent manner [20,35,37,39,42,44,45,46]. The Cyp inhibitor-mediated block was observed for all NS5A genotypes [37,42]. These data strongly suggest that CypA‑NS5A interactions are critical for HCV replication. Altogether these findings provide the first and so far the most tangible initial mechanism of action of Cyp inhibitors that is the prevention of CypA-NS5A contacts.

If this hypothesis is correct, we need to understand how CypA-NS5A interactions govern HCV replication. Key clues came from the Lippens laboratory, which showed that several NS5A prolines, but not all, serve as substrates for *cis-trans* isomerization by CypA [45,47]. Interestingly, the Harris laboratory demonstrated that CypA, via its isomerase pocket, enhances the NS5A RNA-binding capacities [48]. One thus can envision that CypA, by catalyzing the *cis-trans* isomerization of specific proline bonds within NS5A, promotes NS5A binding to viral RNA and facilitates HCV RNA replication. Another possibility is that CypA modulates the contact between NS5A and NS5B, the virally-encoded RNA-dependent RNA polymerase. Previous work showed that NS5A binds directly to NS5B [49]. Moreover, NS5A possesses the capacity to affect the polymerase activity of NS5B [49,50,51]. One thus can envision that CypA, by isomerizing proline bonds in NS5A, influences the ability of NS5A to promote the NS5B polymerase activity. Several studies also suggested that NS5A attenuates the innate immune response towards infection in order to facilitate HCV replication [52]. One cannot exclude the possibility that the *cis-trans* isomerization of NS5A by CypA represents a necessary event for the ability of NS5A to impact the innate response. Therefore, Cyp inhibitors could exert anti-HCV activity through direct (inhibition of recruitment of CypA by virally-expressed proteins impacting the efficiency of viral RNA replication) as well as indirect mechanisms (modulating the innate immune response).

### 2.3. The Clinical Status of Cyclophilin Inhibitors

**Figure 1:**
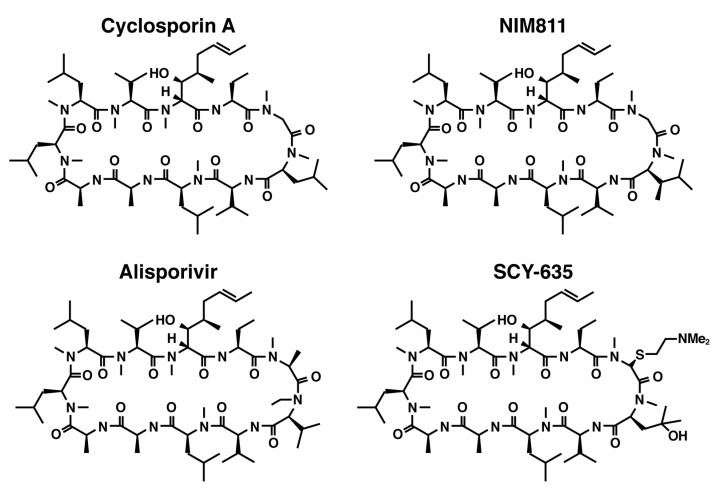
Structural formulas for Cyclosporin A together with three non-immunosuppressive derivatives including NIM811, Alisporivir, and SCY-635.

The clinical utility of CsA-based inhibitors in the treatment of chronic hepatitis C infection has been demonstrated by the introduction of nonimmunosuppressive Cyp inhibitors including NIM811, SCY‑635 and Alisporivir (DEB025) (Figure 1). All three compounds retain the undecapeptide core structure of CsA, but differ from the parent molecule at the 3-sarcosine and 4-*N*-methyl leucine positions. All three compounds are nanomolar inhibitors of the PPIase catalytic activity and form binary complexes with CypA; however, when compared with CsA the binary complexes exhibit greatly diminished affinity for calcineurin. Clinical trials with NIM811 and SCY-635 have been limited to exploratory phase I and II trials enrolling small, relatively well-defined patient populations; therefore, the complete clinical safety profile for these compounds has yet to be determined. In contrast, the safety profile for Alisporivir (DEB025) has been derived from a total patient population consisting of approximately 1,800 patients, which supported the initiation of phase III pivotal clinical testing.

#### 2.3.1. NIM811

The safety, pharmacokinetics, and antiviral activity of monotherapy with NIM811 following single- and multiple-dose administration to treatment-experienced and treatment-naïve adults with chronic genotype 1 hepatitis C infection (n = 72) have been reported [11]. NIM811 was administered orally at total daily doses ranging from 25 mg to 1,200 mg. In general, NIM811 was well-tolerated across all dose groups. Clinically insignificant increases in bilirubin and triglycerides were observed throughout the 14-day treatment period. Thrombocytopenia was observed at total daily doses equal to or greater than 200 mg. No dose reductions were required; however, the authors indicated that the need for dose reduction was mitigated by the relatively short duration of the dosing period. The pharmacokinetics of NIM811 were reported as parameters derived from whole blood concentration *versus* time data; no plasma concentration *versus* time data were reported. Whole blood exposure to NIM811 increased with increasing doses of NIM811. Dose-related, subproportional increases in Cmax and AUC were reported. Suppression of plasma viremia was not observed at any dose level tested as monotherapy including the maximum daily dose of 1,200 mg. Interestingly, rapid normalization of transaminases was observed in the majority of patients who received total daily doses equal to or greater than 100 mg. One additional cohort of patients was added at the completion of the monotherapy phase in order to assess the antiviral activity of NIM811 when administrated in combination with pegylated interferon. Twenty-one patients, all with evidence of relapse following prior treatment with pegylated interferon and ribavirin, were randomly assigned to receive either placebo (n = 10) or NIM811 (n = 11) at a dose of 600 mg given twice daily. Patients in both arms received pegylated interferon (given once weekly on study days 1 and 8 according to the manufacturer’s instructions). Adverse events typically associated with the administration of pegylated interferon (malaise, fever and headache) were reported in the majority of all patients in both arms. Thrombocytopenia (also observed in the monotherapy phase) was more pronounced in patients who received active combination therapy. Whole blood pharmacokinetic parameters in patients who received combination therapy were similar to values reported for patients who received NIM811 monotherapy. At day 14 significant reductions from baseline in viral load were reported for patients who received active combination therapy (2.85 ± 1.02 log10 IU/mL; *p* = 0.0001) when compared to patients who received pegylated interferon plus placebo (0.65 ± 0.77 log10 IU/mL). The authors speculate that the absence of an antiviral effect with monotherapy may be explained on the basis of the sharp *in vitro* dose response of NIM811 in replicon cells coupled with suboptimal clinical dosing; increasing the total daily dose to 1,600 mg (800 mg bid) may be required in order to elicit an antiviral effect as monotherapy. The authors state that the observation of clinical antiviral activity in combination with pegylated interferon was expected on the basis of *in vitro* studies showing potent synergistic effects between NIM811 and interferon in replicon cells. Novartis has discontinued further NIM811 development for Hepatitis C as its antiviral effect was weaker than Alisporivir.

#### 2.3.2. SCY-635

The proof of concept for SCY-635 was established in a phase I clinical trial in adults with chronic hepatitis C infection. [9] Twenty patients with genotype 1 infection received multiple ascending oral doses of SCY-635 or a matching placebo. Total daily doses of SCY-635 equaling 300, 600, or 900 mg/day were administered on a divided schedule three times daily for 15 consecutive days. No evidence of dose-limiting clinical or laboratory toxicity was identified at any dose level. Plasma exposure parameters (Cmax and AUC) increased in a greater than proportional manner as the total daily dose of SCY-635 was increased. Significant distribution of SCY-635 into the plasma compartment was observed in patients receiving the 900 mg daily dose. C8hr plasma concentrations for this cohort remained at or slightly below the replicon-derived EC90 value (463 ng/mL) from Study Day 3 (when the highest plasma concentrations of SCY-635 were achieved) through the completion of treatment on Study Day 15. Treatment with SCY-635 at total daily doses of 300 or 600 mg was associated with minimal, clinically insignificant changes in viral load. In contrast, all 6 subjects who received 900 mg/day exhibited clinically relevant declines in viral load. On Study Day 15 (the final day of treatment) the median viral load reduction for all subjects who received the 900 mg total daily dose was 1.90 log10 IU/mL below baseline; the group mean (± SD) viral load reduction was 2.24 (± 1.74) log10 IU/mL below baseline. Within this dose group, individual maximal declines in viral load ranged from 0.84 to 5.47 log10 IU/mL below baseline. Among subjects receiving 900 mg/day Cmax plasma concentrations and AUC0-8 values exhibited a 2.6- and a 2.9-fold range, respectively, whereas the maximum virological responses differed by approximately 42,000-fold. A post hoc analysis across all dosing groups indicated that subjects with the CC IL28B genotype exhibited the greatest responses to SCY-635 monotherapy. This trend was especially apparent among subjects who received the 900 mg total daily dose. In all subjects who received the 900 mg/day dose there was a concordance between the absorption and disposition of SCY-635 in the plasma compartment and transiently increases from baseline in the plasma protein concentrations of interferon α, interferon λ1 and λ3, as well as the interferon-stimulated gene product, 2'5' oligoadenylate synthetase 1 (2'5' OAS‑1). Pearson correlation coefficients were calculated for the pair-wise comparison of the plasma concentration of SCY-635 together with the plasma concentrations of interferon α, interferons λ1 and λ3, and 2'5' OAS-1. All comparisons yielded values ranging between +0.5 to +1.0 indicating a strong positive correlation for all covariates. Interestingly, the plasma concentration of interferon β, a potent antiviral cytokine, was decreased during dosing with SCY-635; however, the decrease of interferon β did not preclude SCY-635 from exerting antiviral activity at the 900 mg total daily dose especially in patients with the CT and CC IL28B genotypes. The magnitude and the kinetics of the decline in viral load together with the correlation of the antiviral response with host IL28B genotype and the direct demonstration of transient (8 h) increases in the plasma protein concentrations of interferons α, λ1 and λ3, and the interferon-stimulated gene product 2'5' OAS-1 suggest that the SCY‑635-mediated viral replication block (plasma viral load decrease) restores, at least transiently, the innate response. It is important to note that the administration of SCY-635 does not influence interferon and 2'5' OAS-1 plasma levels in non-infected individuals. It is likely that the block of HCV replication in patients treated with SCY-635 allows the innate response to be transiently restored. Indeed, the disappearance of viral proteins, which normally counteract the innate response, should permit its reactivation. Further studies are required to determine whether several weeks of SCY-635 treatment, which should eliminate the virus from the blood, would allow complete reactivation of the innate response. 

#### 2.3.3. Alisporivir (DEB025)

Alisporivir (DEB025) represents the most advanced of the host-targeted Cyp inhibitors now in clinical development. Proof-of-concept was established for Alisporivir when administered as monotherapy to patients who were co-infected with HIV-1 and HCV [10]. Subsequent phase II studies demonstrated the benefit of combination therapy with Alisporivir and pegylated interferon in patients who were naïve to any form of antiviral treatment [53] and combination therapy with Alisporivir together with pegylated interferon and ribavirin in patients with prior evidence of null response [54]. The results of three large randomized phase II studies have been reported. The ESSENTIAL study evaluated the effect of adding Alisporivir to background therapy comprised of pegylated interferon and ribavirin in patients with chronic genotype 1 infection who were naïve to any form of prior treatment [55]. The VITAL-1 study evaluated the effect of Alisporivir administered either as monotherapy, as a 2-drug combination with ribavirin, or with the delayed addition of pegylated interferon and ribavirin to patients with chronic genotype 2 or 3 infection who were naïve to any form of prior treatment [56]. The FUNDAMENTAL study evaluated the effect of adding Alisporivir to background therapy comprised of pegylated interferon and ribavirin in patients with genotype 1 infection and documented evidence of prior nonresponse to interferon-based therapy [57]. Additional phase II and III clinical studies were recently initiated. Unfortunately, a small number of cases reported as acute pancreatitis, including one fatal case in phase 2 and 3 trials in patients treated with Alisporivir in combination with pegylated interferon and ribavirin has placed these clinical studies on partial hold by the FDA. Novartis is currently working with the FDA to resolve their questions. At the time of this writing, there is relatively little publically available information, which can be analyzed in order to evaluate the relationship between the administration of Alisporivir and the development of acute, potentially life-threatening pancreatitis. Unresolved questions concern the possibility that treatment with Alisporivir contributed to pancreatitis by exacerbating underlying conditions associated with individual patient characteristics such as their history of alcohol use, cigarette smoking, or evidence of progressive metabolic disease. Observations of hyperbilirubinemia in previous clinical studies have been explained on the basis of *in vitro* observations demonstrating that Alisporivir is a potent, dose‑related inhibitor of the mrp2-mediated transport of conjugated bilirubin. However, the possibility exists that elevations in bilirubin and the observations of pancreatitis in the phase III program, may have been caused by complications due to other pre-existing undiagnosed conditions such as gall stone disease and are, therefore, unrelated to the administration of Alisporivir. Because these issues remain unresolved the following sections will concentrate only on summarizing published results obtained through the completion of the phase II program.

#### 2.3.4. Exploratory Phase I and II Investigations

Clinical proof-of-concept was established for Alisporivir in patients, who were co-infected with HIV-1 and HCV [10]. Twenty-three adults were randomized to receive either Alisporivir (1,200 mg bid) or a matching placebo for 15 days. The mean maximal reduction from baseline in HCV RNA (expressed as log10 copies/mL) for all individuals, who received Alisporivir was 3.63 compared to 0.73 for individuals, who received placebo. Patients with genotype 3 infection exhibited a mean maximal reduction from baseline in viral load of 4.46 while patients with either genotype 1 or 4 infection exhibited a mean maximal reduction from baseline in viral load of 3.19. These data clearly support the hypothesis that targeting a host factor or inhibiting the recruitment of host factors by virally-expressed proteins confers significant antiviral activity irrespective of the viral genotype. Alisporivir was well absorbed and exhibited a mean plasma Cmax value at steady state of 4,250 ng/mL. Accumulation ratios of 4.9 and 10.1 were calculated for Cmax and AUC0-12, respectively. The most commonly reported adverse events were abdominal pain, feeling hot, vomiting, fatigue, and pyrexia; however, none of these were considered dose-limiting. Fifteen individuals in the Alisporivir group exhibited elevated total bilirubin with 10 individuals becoming icteric, 4 of whom prematurely discontinued treatment. All instances of hyperbilirubinemia were reversible following the cessation of treatment with Alisporivir.

Lower doses of Alisporivir were tested in combination with pegylated interferon in a phase II, 29‑day clinical trial in 90 treatment-naive patients with chronic hepatitis C infection [53]. Patients were randomly assigned to one of five cohorts. In the first four cohorts patients received either placebo or one of three escalating doses of Alisporivir (200, 600, or 1,000 mg) in combination with pegylated interferon. In the fifth cohort patients received Alisporivir monotherapy at a dose of 1,000 mg. In an attempt to evaluate a loading dose strategy, patients received their assigned dose of Alisporivir on a twice daily basis for the first week of treatment. In weeks 2, 3, and 4 patients received their assigned dose of Alisporivir once daily. On day 29 the mean reduction from baseline in viral load was 3.56 log10 IU/mL for patients who received pegylated interferon plus placebo; 3.30 log10 IU/mL for patients, who received 200 mg Alisporivir plus pegylated interferon; and 2.87 log10 IU/mL for patients, who received 1,000 mg Alisporivir monotherapy. Larger declines in viral load were observed in patients who received 600 mg Alisporivir plus pegylated interferon (5.07 log10 IU/mL) and 1,000 mg Alisporivir plus pegylated interferon (5.09 log10 IU/mL). In all treatment groups the declines in viral load were more rapid during the first week of treatment when Alisporivir was administered twice daily. Reductions in viral load among patients, who received Alisporivir monotherapy were more pronounced in patients with genotype 2 or 3 infection when compared with patients with genotype 1 or 4 infection. Headache, nausea, fatigue, and hyperbilirubinemia were the most frequently reported adverse events in patients, who received the 1,000 mg dose of Alisporivir either as monotherapy or in combination with pegylated interferon. The frequency of nausea, fatigue, and hyperbilirubinemia were decreased in patients, who received the two lower doses of Alisporivir.

Alisporivir was assessed in combination with pegylated interferon and ribavirin in genotype 1 null responders [54]. Fifty patients were randomly assigned to one of five treatment arms for a 29-day course of treatment. In treatment arm 1 patients received pegylated interferon plus ribavirin with Alisporivir at a dose of 400 mg given once daily; in treatment arm 2 patients received monotherapy with Alisporivir at a dose of 400 mg given once daily; in treatment arm 3 patients received pegylated interferon with Alisporivir at a dose of 400 mg given once daily; in treatment arm 4 patients received pegylated interferon and ribavirin with Alisporivir at a dose of 800 mg given once daily; and in treatment arm 5 patients received pegylated interferon and ribavirin with Alisporivir given at a dose of 400 mg twice daily for 7 days followed by 400 mg given once daily for the following 22 days. No suppression of viral replication was observed in patients, who received monotherapy with Alisporivir. Alisporivir at a total daily dose of 800 mg when administered in combination with pegylated interferon and ribavirin produced significant suppression of plasma viremia. At day 29 the antiviral activity of Alisporivir was comparable among patients who received Alisporivir when administered as 800 mg qd (2.38 log10 IU/mL decline from baseline) and patients, who received Alisporivir when administered as 400 mg bid (1.96 log10 IU/mL decline from baseline) suggesting that the addition of Alisporivir to pegylated interferon and ribavirin is a viable treatment strategy for patients who exhibit prior null response to interferon-based therapy. 

#### 2.3.5. Large Randomized Phase II Investigations

Table 1 contains a brief summary of the study objectives and major findings for the principal clinical trials that comprise the phase II development program for Alisporivir. 

**Table 1:** Overview of the principal studies in the phase II development program for Alisporivir.

Study	Study Objectives and Patient Population	Total Enrollment	Treatment Assignments	Findings
ESSENTIAL (Final results including SVR24)	Assess the safety and efficacy of Alisporivir when added to Peg IFN/Ribavirin in treatment naive patients with genotype 1 infection ^1^	288	Arm 1: 48 weeks Peg IFN/ Ribavirin plus placebo	Alisporivir was safe and well-tolerated, reversible hyperbilirubinemia was observed beginning during week 1 when patients received the loading dose, increased frequency of neutropenia throughout treatment, Alisporivir improved SVR24 in all groups, only 48 weeks of treatment reached statistical significance compared to control (76% *versus* 55% respectively).
			Arm 2: 48 weeks Peg IFN/ Ribavirin plus Alisporivir 600 mg qd ^1^	
			Arm 3: 48 or 24 weeks Response Guided Therapy Peg IFN/Ribavirin plus Alisporivir 600 mg qd ^1^	
			Arm 4: 24 weeks Peg IFN/ Ribavirin plus Alisporivir 600 mg qd ^1^	
VITAL-1 (Interim results including SVR12)	Safety and efficacy of Alisporivir administered as monotherapy, in combination with ribavirin, or in combination with Peg IFN/Ribavirin in treatment naive patients with genotype 2 or 3 infection ^1,2,3^	385	Arm 1: Alisporivir monotherapy 1,000 mg qd ^1,2,3^	Alisporivir was safe and well-tolerated, hyperbilirubinemia was common among patients receiving Alisporivir regardless of dose. Alisporivir improved SVR12 in all treatment arms, Alisporivir monotherapy or when administered in combination with Ribavirin was curative in a majority of patients who demonstrated RVR; however, most patients did not achieve RVR and met criteria for switching to triple combination therapy.
			Arm 2: Ribavirin plus Alisporivir 600 mg qd ^1,2,3^	
			Arm 3: Delayed addition of Peg IFN/Ribavirin plus Alisporivir 800 mg qd ^1,2,3^	
			Arm 4: Peg IFN/Ribavirin plus Alisporivir 600 mg qd ^1^	
FUNDAMENTAL (Interim results through 12 weeks of treatment)	Safety and efficacy of Alisporivir when combined with Peg IFN/Ribavirin in patients with genotype 1 infection with documented non-response or relapse to prior therapy with Peg IFN/Ribavirin	461	Arm 1: Alisporivir 600 mg qd ^4^	Alisporivir was safe and well-tolerated through 12 weeks of treatment. Most frequently reported adverse events were nausea, neutropenia, and insomnia. Neutropenia was approximately 2-fold greater than control in all arms receiving Alisporivir. Alisporivir increased cEVR in all treatment arms with higher doses exhibiting superior efficacy. Despite the lack of the loading dose in Arm 3, 400 mg bid was superior to 800 mg qd in all analyses.
			Arm 2: Alisporivir 800 mg qd ^4^	
			Arm 3: Alisporivir 400 mg bid	
			Arm 4: Placebo qd or bid* All patients in all treatment arms received background therapy with Peg IFN/Ribavirin*	

^1^ All patients received 7 days of Alisporivir 600 mg bid; on Day 8 patients initiated their randomized dose of Alisporivir. ^2^ Patients exhibiting RVR (<25 IU/mL) received their originally randomized regimen throughout the remainder of the 24-week treatment period. ^3^ Patients with viral load ≥25 IU/mL at Week 4 switched treatment at Week 6 to triple therapy containing Alisporivir 600 mg qd plus Peg IFN/ Ribavirin for the remaining 18 weeks. ^4^ Patients in Arms 1 and 2 received 7 days of loading dose treatment with Alisporivir 600 mg bid. On Day 8 patients initiated treatment with their randomized dose of Alisporivir.

#### 2.3.6. ESSENTIAL

Final results for the ESSENTIAL study have been reported [55]. The objectives of the study were to evaluate the safety and efficacy of Alisporivir when added to a background regimen comprised of pegylated interferon and ribavirin when administered to treatment-naïve patients, who were chronically infected with genotype 1 virus. The primary endpoint was to compare the proportion of patients in each arm who achieved SVR24 using 10 IU/mL at the limit of detection for the plasma concentration of HCV-specific RNA. Two hundred and eighty-eight patients were randomly assigned to participate in 1 of 4 treatment arms. Patients in all arms received background therapy with pegylated interferon and ribavirin. In treatment arm 1 patients received placebo once daily (n = 73) for a total duration of 48 weeks. In arms 2, 3, and 4 patients received active treatment with Alisporivir once daily at a dose of 600 mg; however, during week 1 all patients in arms 2, 3, and 4 received Alisporivir administered twice daily at a dose of 600 mg (*i.e.*, 1,200 mg total daily dose) as part of a loading dose strategy. Beginning on day 8 all patients received a reduced dose of Alisporivir. Study drug was administered once daily at a dose of 600 mg. This treatment schedule was maintained through the end of the dosing period. In treatment arm 2 patients received Alisporivir in combination with background therapy (n = 72) for 48 weeks; in treatment arm 3 Response Guided Therapy was used to determine the duration of treatment with Alisporivir in combination with background therapy (n = 71). Within arm 3, patients who demonstrated a rapid virological response were eligible to receive 24 weeks of treatment. Patients, who did not demonstrate a rapid virological response would continue treatment for 48 weeks; in treatment arm 4 patients received Alisporivir in combination with background therapy (n = 72) for 24 weeks. A planned follow up evaluation occurred 24 weeks after the end of treatment in all arms regardless of the length of the treatment period.

Alisporivir was well-tolerated in all arms through the 24- and 48-week treatment periods. There were no deaths and the frequencies of serious adverse events and discontinuations were similar across all arms. In all arms where Alisporivir was administered, there was an increased frequency of some laboratory abnormalities including total bilirubin. In the control arm the frequency of hyperbillirubinemia was 1.4%; whereas, the frequency of hyperbillirubinemia was 32.9, 25.4, and 41.7% in arms 2, 3, and 4, respectively. Hyperbilirubinemia was predominantly associated with the first week of therapy during which time patients received Alisporivir at a total daily dose of 1,200 mg. Total bilirubin values decreased beginning in week 2 when the total daily dose of Alisporivir was decreased to 600 mg; however, total bilirubin values remained elevated through the end of the treatment period. Total bilirubin returned to pretreatment values at the 12-week follow-up evaluation. The frequency of grade 3 and 4 neutropenia was 29.2% in the control arm. Although not reaching statistical significance, the frequency of neutropenia was greater than control in all arms containing Alisporivir. Values were 43.1, 38.0, and 33.3% for arms 2, 3, and 4, respectively. The frequency of thrombocytopenia was somewhat increased in patients in arm 3 who received 24 weeks of response‑guided therapy with Alisporivir (7.0%) in comparison to patients in arm 1 who received control therapy (1.4%).

The addition of Alisporivir improved SVR24 in all arms relative to control therapy. SVR24 for the control arm was 55%. SVR24 for arm 2 (48 weeks of Alisporivir), arm 3 (24 or 48 weeks of Alisporivir RGT), and arm 4 (24 weeks of Alisporivir) was 76, 69, and 53%, respectively, with only arm 2 reaching statistical significance (*p* = 0.008) when compared to control. This result was achieved despite the observation that the frequency of the CC IL28B allele was 33% in the control arm and 19% in arm 2. Among patients with the CC IL28B allele, end-of-treatment responses were high (100% of patients exhibited undetectable HCV plasma RNA) and were maintained at the 24-week follow-up (100% SVR24) for the subgroups of patients who received either a planned treatment duration of 48 weeks or 24 weeks of RGT with Alisporivir. End-of-treatment responses declined at the 24-week evaluation for CC patients who received control treatment or a planned 24-week treatment with Alisporivir. The benefit of adding Alisporivir to background therapy was apparent among patients with the TT IL28B genotype. SVR 24 for patients with the TT IL28B genotype who received control therapy was 17%. SVR24 increased to 33% in patients who received 24 weeks of treatment with Alisporivir, 62% in patients who received 48 weeks of treatment with Alisporivir, and 73% in patients who received Alisporivir RGT. RVR was improved in all Alisporivir treatment groups relative to control.

The frequency of viral breakthrough was low (approximately 2.8%) in patients receiving Alisporivir. Viral breakthrough was observed only in patients with the CT or TT IL28B genotype and was associated either with reduction in dose or cessation of treatment with ribavirin or pegylated interferon or with suboptimal plasma concentrations of Alisporivir (reflecting either low individual oral bioavailability or poor compliance with the prescribed treatment). Near full-length HCV genomic RNA was sequenced at Baseline and at the time of breakthrough from 6 patients. The D320E mutation, which is located in the C-terminal portion of domain II in the NS5A protein, was detected in 3 of the 6 patients. D320E was detected in only one patient at the time of breakthrough whereas it emerged in the remaining two patients approximately 6 weeks and 18 weeks after the initial breakthrough was observed. Phenotypic analysis of drug sensitivity using replicons constructed with patient-derived NS5A sequences indicates that the D320E substitution is associated with causing a relatively modest (approximately 3-fold) increase in the EC_50_ value against Alisporivir. These results suggest that other mutations in concert with D320E may account for viral breakthrough during treatment with Alisporivir. The authors concluded that the addition of 48 weeks of Alisporivir at a total daily dose of 600 mg to pegylated interferon and ribavirin improves SVR24 in treatment-naïve patients infected with genotype 1 virus.

#### 2.3.7. VITAL-1

The initial results (representing 12 weeks of follow-up after the completion of treatment for the majority of all enrolled patients) of the VITAL-1 study were recently reported and are summarized here [56]. The objectives of the study were to evaluate the safety and efficacy of Alisporivir when administered either as monotherapy, as part of a 2-drug combination with ribavirin, or as part of a 3‑drug regimen with the delayed addition of pegylated interferon and ribavirin for the treatment of patients who were chronically infected with genotype 2 or 3 virus and were naïve to prior therapy with pegylated interferon and ribavirin. Three hundred and eighty-five patients were randomly assigned to participate in 1 of 5 treatment arms. In treatment arm 1 patients received Alisporivir once daily as monotherapy at a dose of 1,000 mg (n = 83); in treatment arm 2 patients received Alisporivir once daily at a dose of 600 mg in combination with ribavirin (n = 84); in treatment arm 3 patients received Alisporivir once daily at a dose of 800 mg in combination with ribavirin (n = 94); in treatment arm 4 patients received Alisporivir once daily at a dose of 600 mg in combination with pegylated interferon (n = 84); in arm 5 patients received control therapy comprised of pegylated interferon and ribavirin (n = 40). All patients in all arms received 7 days of loading dose treatment with Alisporivir at a dose of 600 mg given twice daily (*i.e.*, 1,200 mg/day for the first 7 days of treatment) after which patients initiated treatment with their randomized dose of Alisporivir. The total duration of treatment for all arms was 24 weeks with planned follow-up evaluations at weeks 36 (to assess SVR12) and 48 (to assess SVR24). Viral load was assessed at week 4 for the determination of rapid virological response rates for all treatment arms and to determine if a modification of the originally randomized treatment was necessary. If a patient demonstrated a rapid virological response with viral load <25 IU/mL then the patient would remain on their originally assigned treatment; however, if a patient exhibited viral load at week 4 which was in excess of 25 IU/mL, then they would switch at the beginning of week 6 to treatment with Alisporivir administered once daily at a dose of 600 mg given in combination with pegylated interferon and ribavirin and maintain that regimen for the following 18 weeks through the end of treatment at week 24.

Alisporivir was well-tolerated in all arms through the 24-week treatment period. More serious adverse events and more discontinuations were reported in the arms containing Alisporivir; however, the majority of these events appeared to be unrelated to Alisporivir. As anticipated, the interferon-free arms were associated with a decreased frequency of adverse events such as psychiatric disorders, fatigue, nausea, influenza-like illness *etc.*, when compared to arms containing pegylated interferon. There was also a decreased frequency of laboratory abnormalities including anemia, neutropenia, and thrombocytopenia in the interferon-free arms. The majority of patients, who received Alisporivir experienced hyperbilirubinemia, which was detected at the week 1 evaluation and was maintained through the end of treatment at week 24. The majority of patients exhibited values that exceed their pretreatment baseline values, but remained below 3× ULN; however, some patients exhibited values that exceeded 5× ULN. Patients exhibited mixed hyperbilirubinemia. Mechanistically this was attributed to *in vitro* observations, which indicate that Alisporivir inhibits the uptake transporters OATP1B1 and 1B3 (leading to elevated indirect bilirubin) and the efflux transporter mrp 2 (leading to elevated direct bilirubin).

SVR12 was higher among the four treatment arms that included Alisporivir in comparison to the control arm containing pegylated interferon and ribavirin when calculated both on an Intent-to-Treat basis and on an Efficacy-Evaluable basis. For the Intent-to-Treat population SVR12 values were 81, 83, 81, 77, and 58% for arms 1, 2, 3, 4, and 5, respectively. For the Efficacy-Evaluable population SVR12 values were 91, 91, 94, 86, and 74% for arms 1, 2, 3, 4, and 5, respectively. The proportion of patients, who achieved RVR was 29, 37, and 42% for arms 1, 2, and 3, respectively. Among patients, who demonstrated RVR 82, 93, and 91% of patients in arms 1, 2, and 3 achieved SVR12 indicating that Alisporivir administered either as monotherapy or in combination with ribavirin is a viable all-oral treatment strategy for treatment-naïve patients with chronic genotype 2 or 3 infection who demonstrate empiric evidence of a robust virological response after 4 weeks of treatment; however, the majority of all patients in arms 1 through 4 did not achieve RVR and therefore met the predetermined criterion for switching at week 6 to a regimen comprised of Alisporivir (600 mg qd) plus pegylated interferon and ribavirin. After switching, patients remained on this regimen for 18 weeks until the end of treatment. SVR12 values among patients who switched to triple therapy because they did not achieve RVR were 94, 92, and 96% for arms 1, 2, and 3, respectively, indicating that the delayed addition of pegylated interferon and ribavirin by 6 weeks to a background regimen containing Alispsorivir improves viral clearance in patients with chronic genotype 2 or 3 infection who do not achieve RVR.

#### 2.3.8. FUNDAMENTAL

The initial results (representing a planned interim analysis for data collected after 12 weeks of treatment) of the FUNDAMENTAL Study were recently reported and are summarized here [57]. The objectives of the study were to evaluate the safety and efficacy of Alisporivir when administered in combination with pegylated interferon and ribavirin for the treatment of patients with chronic infection with genotype 1 virus with a documented history of relapse or nonresponse to prior treatment with pegylated interferon and ribavirin. Four hundred and sixty-one patients were randomly assigned to participate in 1 of 4 treatment arms. In treatment arm 1 patients received Alisporivir once daily at a dose of 600 mg (n = 121); in treatment arm 2 patients received Alisporivir once daily at a dose of 800 mg (n = 117); in treatment arm 3 patients received Alisporivir at a total daily dose of 800 mg administered as 400 mg bid (n = 109); in treatment arm 4 patients received placebo once or twice daily (n = 114). All patients in all groups received background treatment with pegylated interferon (180 µg/week) and ribavirin (1,000 or 1,200 mg/day based on BMI). All patients in arms 1 and 2 received 7 days of loading dose treatment with Alisporivir at a dose of 600 mg given twice daily (*i.e.*, 1,200 mg/day for the first 7 days of treatment) after which patients initiated treatment with their randomized dose of Alisporivir. The total duration of treatment for all arms was 48 weeks with planned follow-up evaluations at weeks 60 (to assess SVR12) and 72 (to assess SVR24). The primary endpoint for the planned interim analysis at week 12 of treatment was a comparison of the proportion of patients in each treatment arm who achieved cEVR (defined as undetectable serum HCV RNA below the limit of quantitation of 25 IU/mL). cEVR rates were also evaluated based on the extent of prior response and based on IL28B genotype (CC *versus* CT/TT). Viral load was assessed at week 4 for the determination of rapid virological response rates for all treatment arms

Alisporivir was well-tolerated at all dose levels throughout the first 12 weeks of treatment. The frequency of serious adverse events and discontinuations due to adverse events were similar across the experimental and control arms. The most frequently reported adverse events were nausea, neutropenia, and insomnia. In all experimental arms the proportion of patients experiencing neutropenia was approximately 2-fold greater than control (32, 28, 35, and 16% for arms 1, 2, 3, and 4, respectively).

The addition of Alisporivir increased the proportion of patients, who achieved cEVR with 46.4, 61.1, 71.3, and 32.7% of all patients in arms 1, 2, 3, and 4 meeting the primary endpoint, respectively. Although the total daily nominal doses were identical in arms 2 and 3 (800 mg/day), Alisporivir administered as 400 mg bid (arm 3) yielded a higher proportion of patients who achieved cEVR in comparison to 800 mg qd (arm 2). Arm 3 also demonstrated consistently greater decreases in HCV‑specific RNA over time in comparison to Arms 1 and 2 and in comparison to control. For relapsers, all doses of Alisporivir yielded higher proportions of patients achieving cEVR with values of 62.5, 77.6, 72.9, and 51.9 for arms 1 through 4, respectively. The same trends were apparent for nonresponders with arms 2 and 3 showing highly statistically significant differences in comparison to control (arm 2, 47.5% cEVR, *p* < 0.0001 *versus* control 14.3% cEVR; arm 3, 70% cEVR, *p* < 0.00001 *versus* control). The 400 mg bid dose (arm 3) was particularly effective among null nonresponders and partial nonresponders and yielded similar results (69.7 and 68.0%, respectively) between these two subgroups. The 400 mg bid dose was most effective in the null nonresponder subgroup and increased the proportion of patients achieving cEVR in comparison to placebo (69.7 *versus* 11.1%). Viral breakthrough was low across all groups and was 3.6, 3.7, 1.8, and 2.7% for groups 1 through 4, respectively, further reflecting the superior efficacy of the 400 mg bid dose. The authors concluded that the addition of Alisporivir to a background regimen comprised of pegylated interferon and ribavirin could represent a new option for difficult-to-treat patients, especially those with genotype 1 infection with demonstrated prior nonresponse to interferon-based regimens. This study is currently in progress and further interim analyses are planned for the week 24 time point.

## 3. Conclusion

A growing body of evidence now confirms that Cyps, and in particular CypA, are essential host cofactors whose expression is necessary in order to support HCV-specific RNA replication. By extension, nonimmunosuppressive Cyp inhibitors including NIM811, SCY-635, and Alisporivir have come under intense scrutiny in order to vet host CypA as a target for antiviral chemotherapy and for their use as chemical probes to understand the basic biology of HCV infection and to evaluate their use as potential therapeutic agents.

Cyp inhibitors may act through multiple mechanisms and exert antiviral activity through direct as well as indirect pathways. Direct effects of Cyp inhibitors include their potent inhibition of peptidyl‑prolyl isomerase catalytic activity associated with the broad family of Cyps and their ability to promote the dissociation of multi-protein complexes formed between CypA and viral nonstructural proteins, in particular, NS5A. Dissociation of the CypA/NS5A complex may, in turn, decrease the affinity of NS5A for viral RNA leading to decreased efficiency of viral replication. Regarding possible indirect effects, recent evidence from clinical studies with SCY-635 monotherapy indicate that effective treatment was associated with a transient increased expression of multiple antiviral cytokines including interferons α, λ1 and λ3 and the interferon-stimulated gene product 2'5' OAS-1. This transient increase of components of the interferon response may facilitate the clearance of the virus. Further work is required to determine whether or not the transient reactivation of the innate response by Cyp inhibitor administration is simply due to a block in viral replication or to an unknown mechanism. It is unlikely that Cyp inhibitors alone trigger the activation of the innate response since no Cyp inhibitors effect was observed in non-infected individuals [9].

Clinical studies conducted to date, and especially those conducted with Alisporivir, have established that the addition of a potent Cyp inhibitor to a background regimen comprised of pegylated interferon and ribavirin improves SVR in multiple patient groups including treatment-naïve patients with genotype 1 infection, nonresponders/relapsers with genotype 1 infection, and treatment-naïve patients with genotype 2 or 3 infection. With the doses of ALV and RBV used in the VITAL-1 study, the proportion of patients that that achieved early HCV clearance (RVR—week 4) was about 40%, which is relatively low, however of those cases that had RVR and continued with ALV+RBV—92% achieved SVR24. These data substantiate the concept that Cyp inhibitors are effective components of all-oral treatment strategies and emphasize the point that future directions for clinical evaluation should focus on determining the optimal all-oral treatment strategy for regimens that include Cyp inhibitors. Basic clinical studies would include determining drug interaction profiles between Cyp inhibitors and approved protease inhibitors as well as emerging investigational agents such as nucleoside-based polymerase inhibitors. Other approaches should include building on existing data in order to determine biochemical correlates of antiviral response (such as changes in basal levels of endogenous antiviral cytokines and interferon-stimulated genes) that may be used to identify patient populations that will derive the greatest benefit from treatment regimens that include Cyp inhibitors. 
